# The Factors Influencing Older Adults’ Decisions Surrounding Adoption of Technology: Quantitative Experimental Study

**DOI:** 10.2196/39890

**Published:** 2022-11-23

**Authors:** Jerad Moxley, Joseph Sharit, Sara J Czaja

**Affiliations:** 1 Division of Geriatrics and Palliative Medicine Center on Aging and Behavioral Research Weill Cornell Medicine New York, NY United States; 2 Department of Industrial and Systems Engineering University of Miami Miami, FL United States

**Keywords:** aging, technology, design, older adult, technology, application, independence, relationship, adopt, transportation, leisure, health, learning, adoption, cognition, cognitive, willingness, design, marketing, consumer, mobile phone

## Abstract

**Background:**

The rapid diffusion of technology apps may support older adults’ independence and improve the quality of their lives. Models for predicting technology acceptance in older adults are sparse, based on broad questions related to general technology acceptance, and largely not grounded in theories of aging.

**Objective:**

This study aimed to use a mixed methods approach involving 5 technologies to comprehensively assess the causal relationships among factors that influence older adults’ willingness to adopt the technologies.

**Methods:**

In total, 187 men and women aged 65 to 92 years participated in the study. Participants were given presentations on 5 different technologies spanning domains that included transportation, leisure, health, and new learning and provided ratings of each technology on various measures hypothesized to influence adoption. They were also administered other instruments to collect data on their actual and self-assessed cognitive abilities, rates of discounting of the technologies with respect to willingness to invest time to attain higher skills in the technologies, general technology experience, and attitudes toward technology. We used the machine learning technique of k-fold cross-validated regressions to select variables that predicted participants’ willingness to adopt the technologies.

**Results:**

Willingness to adopt technologies was most impacted by 3 variables: perceived value of the technologies (β=.54), perceived improvement in quality of life attainable from the technologies (β=.24), and confidence in being able to use the technologies (β=.15). These variables, in turn, were mostly facilitated or inhibited by the perceived effort required to learn to use the technologies, a positive attitude toward technology as reflected in the optimism component of the technology readiness scale, the degree to which technologies were discounted, and the perceived help needed to learn to use the technologies.

**Conclusions:**

Our findings demonstrate that participants’ willingness to adopt technologies is mainly determined by perceptions of 3 aspects of the technologies; these aspects possibly mediate many relationships with willingness to adopt. We discuss the implications of these findings for the design and marketing of technology products for older consumers.

## Introduction

### Background

New technologies are diffusing into everyday life at an extraordinary pace. These technologies span domains that include health and wellness, communication and socialization, transportation, entertainment, lifelong learning, and home support and may be found in different forms such as websites, mobile apps, wearable devices, and consoles. Importantly, many existing and emerging technologies may afford older adults with opportunities for enhanced independence, quality of life [1], and more successful aging [2-5] by promoting the maintenance of mental and physical health and life-engagement activities and, more generally, the continuation of adaptation to age-related changes over the life span [6]. However, older adults consistently adopt technology at lower rates compared with younger age groups [7-9], which compromises their ability to derive benefits offered by technology.

### General Models of Technology Acceptance

Given the broad and important implications of technology use, even beyond older adults, several models have been developed and refined with the purpose of delineating factors that predict technology adoption in the general population. A widely cited early model is the Technology Acceptance Model (TAM) [10,11]. It posits that the use of a technology system is predicated by an individual’s motivation to use it, which depends on 3 variables: the perceived usefulness of the technology, the perceived ease of use of the technology, and overall attitude toward using the technology.

Various modifications of the TAM [12] led to TAM 2 [13], which largely focused on technology use in the workplace. It proposes additional variables that influence the perceived usefulness of a technology, such as job relevance and output quality. Another widely cited model for technology acceptance is the Unified Theory of Acceptance and Use of Technology (UTAUT) [14], which coalesces data from 8 prior technology adoption models that consider the roles of constructs such as social factors, job fit, subjective norms, perceived ease of use and usefulness, self-efficacy, and attitudes toward behavior. The UTAUT allows for age to interact with every relationship in the model.

The UTAUT model also posited 4 additional factors important in the behavioral intention to adopt technology: facilitating conditions, social influence, effort expectancy, and performance expectancy. However, the data that the UTAUT and its precursor models were based on were largely derived from students and the concern for people in work situations who were reticent to adopt workplace technologies that could potentially benefit themselves and their employers. As stated by these authors, “UTAUT thus provides a useful tool for managers needing to assess the likelihood of success for new technology introductions and helps them understand the drivers of acceptance to proactively design interventions (including training, marketing, etc) targeted at populations of users that may be less inclined to adopt and use new systems” [14]. In addition, these models were not explicitly tested with older age groups and thus did not specifically address factors associated with older populations. To this end, the UTAUT model has been extended with new constructs such as privacy and to new populations [15].

### Models of Technology Acceptance Specific to Older Adults

The Senior TAM (STAM) [16,17] also attempts to address these issues. On the basis of the TAM and UTAUT modeling frameworks, the STAM was developed to predict the acceptance of general technology by Hong Kong Chinese older adults through the inclusion of several factors. Although both the TAM and UTAUT propose a causality flow whereby a set of constructs causes another set of constructs, which in turn causes the use of technology, the STAM differs from the TAM and UTAUT in that the causal specifications are much broader.

For example, in the TAM, the direct effect of perceived ease of use on the behavioral intention to use technology is predicted to be influenced by attitudes about the technology. In contrast, the STAM model found no support for a direct effect of perceived ease of use (or of perceived usefulness) on the actual use of technology. Although both perceived ease of use and perceived usefulness predicted attitudes toward the use of technology, attitudes did not significantly predict actual use. Instead, the STAM found a broad array of variables, including gerontechnology self-efficacy, gerontechnology anxiety, facilitating conditions (knowledge, guidance, and support from other people), health and ability characteristics, social relationships, and attitudes toward life and satisfaction, to have a direct effect on the actual use of technology. In addition, almost every one of these predictors had a direct effect on perceived ease of use, perceived usefulness, or both variables. However, the general nature of how causality is specified in this model implies that it is difficult to refute [18].

It should also be noted that the development of the STAM [16] was based on the outcome measure of how many technologies respondents had used in the previous 12 months. Therefore, in contrast to other models of technology adoption, the outcome was retrospective and not concurrent or prospective. This raises the possibility that the conclusions that formed the STAM model may have been due to differences in older adults' retrospective technology use rather than based on their concurrent or prospective attitudes toward adopting technology.

More recently, Harris and Rogers [19] developed a health care TAM based on older adults with hypertension. In total, 23 older adults were interviewed, and the interview transcripts were analyzed to identify factors that were frequently mentioned for the consideration of use of each of 3 health care technologies: a blood pressure monitor, an electronic pillbox, and a multifunction robot. Perceived ease of use, perceived usefulness, facilitating conditions, and social influences—4 predictors commonly used in the theoretical models of technology acceptance associated with the TAM—were assumed to be the primary drivers of behavioral intentions to adopt technology. The qualitative analysis revealed that a host of other factors could impact these predictors, including perceived need, privacy and trust in the technology, familiarity, and advice acceptance.

### Study Objectives

This study focused on deriving a more comprehensive understanding of the factors underlying and interactively influencing older adults’ willingness to adopt technology within a concurrent context. Specifically, participants provided appraisals based on exposure to actual technologies, in contrast to eliciting responses from older adults regarding acceptance of “general technology” [17] through broadly based questions. We used a mixed methods experimental approach referred to as the Technology Assessment Procedure (TAP), which provided study participants with some requisite exposure to a set of specific technologies.

The focus, however, was not on these specific technologies; the technologies selected were exemplars of a potentially large number of existing and future technologies across several life domains. In selecting an exemplar set of representative technologies that could be used to experimentally investigate predictors of willingness to adopt technology, our objective was to ensure that the technologies spanned different domains (eg, transportation, health and wellness, and lifelong learning), spanned different forms (eg, mobile apps and websites), and were potentially relevant because of their ability to improve the quality of life of older adults [1]. Simultaneously, we wanted to ensure that it was feasible and comfortable for our older study participants to evaluate the selected technologies during a single experimental session, which we validated during pilot studies.

Using the TAP methodology, we obtained participants’ ratings for each of the technologies on measures such as perceived value, improvements to quality of life, confidence in the ability to use the technology, concerns for privacy, perceived effort needed to learn the technology, and perceived help needed from family and friends to use the technology. As part of our methodology, we also examined cognitive ability measures, subjective ability measures based on self-assessments, participants’ discounting behavior, and general technology experience.

The primary goal of this study was to determine, using a concurrent framework whereby participants’ appraisals are provided within the context of actual technologies that are presented to them, those variables that are most critical in directly impacting the willingness to adopt these technologies. We also sought to establish possible mediative roles by identifying facilitating and inhibitory influences on these variables. As discussed in the ensuing section, a number of these variables were derived based on our prior findings regarding older adults’ use of technology and from cognitive aging theory. Understanding the interplay of these influences is critical, both to our theoretical knowledge concerning older adults and technology adoption and for providing a blueprint for more effective design of technology products for older populations and strategies for marketing these products to older consumers.

### A Modeling Framework: Predicting Older Adults’ Willingness to Adopt Technologies

In total, 5 exemplar technologies were targeted in this study. For each of these technologies the variables examined, using the TAP methodology, included participants’ self-appraisals of the willingness to adopt the specified technology, the value or importance of the technology, the (mental) effort needed to learn and master the technology, the confidence in one’s ability to learn and master the technology, the degree to which help would be needed from family and friends to learn and master the technology, the degree to which the technology is perceived as improving one’s quality of life, concerns with issues of privacy and trust associated with the technology, and the willingness to pay for the technology.

In addition, other factors related to willingness to adopt technology that could be impacted by age were considered. These included perceived health status, openness to and readiness to take on technology, the degree to which the technology is discounted because of the investment of time needed to obtain skills on it, self-assessment of one’s cognitive abilities, cognitive abilities (based on cognitive tests), experience using computer technologies, knowledge and skills related to the use of technology, and the degree and nature of support available from family or friends for learning or using the technology.

The machine learning technique of k-fold cross-validated regressions was used to select variables that directly predicted participants’ willingness to adopt the technologies. We then used multiple regression analyses to determine the best predictors of the variables selected by k-fold cross-validated regressions. This technique is well suited to this study, as data were collected on many variables, including those that are highly correlated with each other. In addition, the models of technology adoption reviewed serve mostly as frameworks for describing the types of factors that likely influence technology adoption and are not intended to be rigorous enough to be the basis for a fully confirmatory model. We hypothesized that the perceived value of the technology, based on empirical studies involving older adults [1], would be a strong predictor of its adoption. We also predicted that confidence in one’s ability to learn the technology, which relates to the construct of self-efficacy in technology acceptance-based models (eg, STAM), and the degree to which the technology is perceived as improving one’s quality of life would be strong predictors of willingness to adopt technology based on our past findings [1].

In addition, the cognitive effort perceived to be needed to learn and master a technology was expected to have an inhibitory influence on the intention to adopt technologies, given the general tendency for people to minimize expenditure of cognitive effort [20] and the reductions with age in “metacognitive beliefs” [21] concerning cognitive capabilities. In addition, from the perspective of learning and skill acquisition [22,23], given that older adults learn new material more slowly than younger adults, the possible requirement for a greater investment of mental effort for older adults to learn the technology may inhibit their intention to adopt it. Willingness to learn new things, which is related to the trait known as “openness to experience” and to the construct of technology readiness, was also expected to indirectly influence the willingness to adopt technologies as it typically diminishes with aging [24].

Another age-related factor that we believe would influence the willingness to adopt technologies is the extent to which rewards received later in time are discounted. For decisions based on more realistic (ie, not hypothetical monetary) types of future rewards, Melenhorst [25] found increased discounting with age, which is consistent with economic perspectives on aging and discounting [26]. However, Sharit et al [27] found that older adults discounted less with increasing age when rewards consisted of attaining greater skills on technologies. In this context, the willingness to invest more time than someone else to achieve the same reward (ie, level of skill in a technology) would reflect lesser discounting, similar to the willingness to wait a longer period than someone else to accrue the same amount of monetary reward. We hypothesized that lower discounting would imply greater willingness to adopt the technologies, especially if the technologies are perceived to provide improvements to the quality of life.

There may be concerns with privacy that older adults harbor, which may depend on the technology, for instance, apps that are designed to support health or financial management [1]. In addition, willingness to pay for the technology (for those technologies or apps for which such costs apply) may also influence the intention of older adults to adopt technologies [28]. The hypothesized effects of these and some additional variables are presented in Table 1.

Through the identification of variables that directly influence the willingness to adopt the technologies presented to participants and determining their possible facilitating and inhibitory indirect influences and thus possible mediating roles, as indicated in some of the examples considered above, we hoped to develop a better understanding of the interplay of influences on the adoption of technologies by older adults. Overall, the goal of this study was to measure these variables, test their hypothesized influences, and ultimately derive an efficient model that reliably captures, across a range of technologies relevant to older adults, the dynamic interplay of factors governing the willingness of older adults to adopt technologies.

**Table 1:** Hypothesized effects of increases in selected study variables on willingness to adopt the technologies.

Variable	Expected effect
Perceived value	Positive
Confidence in ability to use the technology	Positive
Perceived ability for the technology to improve quality of life	Positive
Perceived help needed to learn the technology	Negative
Perceived cognitive effort needed to learn the technology	Negative
Technology readiness	Positive
Discounting of time willing to invest to learn the technology	Negative
Concerns with privacy	Negative
Willingness to pay for the technology	Positive
Self-assessment of abilities	Positive
General technology experience	Positive
Availability of technology assistance	Positive

## Methods

### Participants

Participants were recruited from 2 large US cities through advertisement in local media and newsletters, interactions with agencies serving older adults, and participant registries. Interested participants completed an initial telephone interview that assessed basic eligibility, which included being ≥65 years of age; able to read and understand English at the sixth grade level; having no problems related to hearing (with correction), vision (at least 20/70 with correction), or arthritis that would impair their ability to write or use a laptop computer (only 2 people were excluded based on this criterion); being noncognitively impaired as measured by the Telephone Interview for Cognitive Status instrument [29], with cutoff scores adjusted for age and education (eg, for people between 70 and 79 years of age, a minimal score of 29 was required for those with less than a high school education, and a minimal score of 31 was required for those with at least a high school education); and having no experience with any of the 5 technologies presented in the study.

### Ethics Approval

The participants provided written informed consent and were compensated US $40 (and any parking expenses) for their participation. The Institutional Review Boards of the University of Miami and the Weill Cornell Medicine approved the study (approval number 1808019538).

### Procedure

The experimental procedure used a modified version of a mixed methods data collection procedure referred to as the TAP. This method [1] involves the following: (1) presenting study participants with in-depth overviews of various technologies; (2) after each technology presentation, completing a questionnaire to rate the technology on various criteria related to its adoption; (3) completing additional questionnaires and other assessments intended for complementing the data on technology ratings; and (4) participation in postpresentation focus groups. In this study, the focus group feature of TAP was not implemented.

Each study session involved groups of 2 to 4 people. Participants were introduced to the study, provided written informed consent, and then individually administered a demographics questionnaire, the Wide Range Achievement Test [30] to assess literacy, and a vision test. Participants who did not meet the inclusion criteria were compensated US $10 for their time. Participants who met the inclusion criteria proceeded, in a sequential order, through the ensuing session steps, with rest breaks given to them between several of these steps.

The assessment typically took approximately 4 hours. Participants were provided with snacks and drinks during the sessions, a formal break after the technology ratings were completed, and restroom breaks as needed. Although the order in which the technologies were presented and rated was randomized, the order of the instruments did not vary. As the most important measure for this study was the technology ratings, these ratings were completed first. We believed, based on prior experience, that cognitive testing would constitute the most taxing aspect of the study for the participants; thus, these measures were collected last to lessen the effect of fatigue on the other components of the study. Furthermore, as the study was not concerned with the level of the cognitive measures with respect to classifying cognitive status or abilities but rather with examining the potential impact of individual differences, this approach seemed better than adding an additional design factor whereby some participants completed the cognitive measures at the beginning of the study and were more fatigued before doing different parts of the study than other participants.

### Technology Presentations and the Technology Ratings Questionnaire

#### Overview

**Figure 1:**
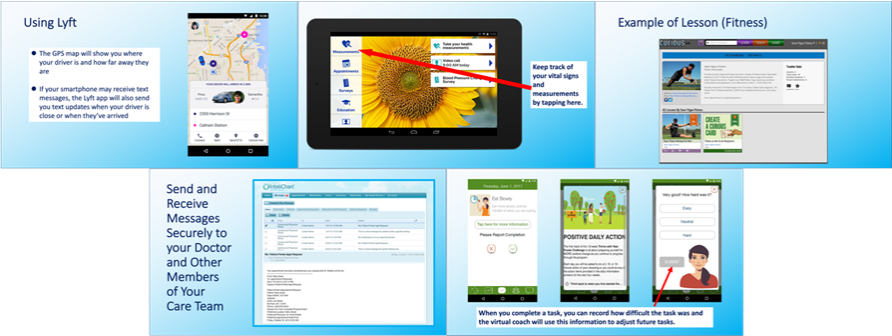
Example of slides used in the presentations of the technologies to participants going clockwise from the top left: Lyft, eCareCompanion, Curious, Fittle (bottom right), and InteliChart.

Participants as a group were shown PowerPoint presentations on 5 technologies in a predetermined random order to minimize order effects. The five technologies were (1) Lyft, a ride-sharing app; (2) eCareCompanion, an app that allows sharing of health information with your care team, tracking of health tasks, and optional devices to measure vital statistics; (3) Curious, a website dedicated to providing lessons for lifelong learners on a variety of topics; (4) InteliChart, a patient portal that allows an individual to view medical charts, schedule appointments, and manage other aspects of health care; and (5) Fittle, an app that uses an internet-based coach to help people meet health and fitness goals. Each presentation lasted for approximately 10 minutes. Participants were allowed to ask clarifying questions about each technology; however, discussion among the participants was not permitted. Figure 1 shows examples of the slides used in the presentation of the technologies.

Following the presentation of each technology, participants completed a technology rating questionnaire in which they rated the technology on various criteria using a Likert-type 9-point scale (except for the willingness-to-pay criterion), with verbal descriptors provided for the 2 endpoints and the 3 intermediary points on the scale. After the presentations on all 5 technologies were completed, a summary of the 5 technologies was presented, and participants were able to review their ratings and make changes if desired. The participants rated each technology based on the following criteria.

#### Willingness to Adopt the Technology

How willing are you to adopt the technology that was just presented to you? In other words, how willing are you to “take it up” and start using it (1 being completely unwilling, 5 being somewhat willing, and 9 being completely willing)? This was the primary dependent variable in the analyses. We also measured willingness to adopt using paired comparisons within the Analytic Hierarchy Process [31,32]. Although the paired comparisons measure validated our willingness to adopt measures, we chose not to use this measure in our analyses because it was highly correlated with the willingness to adopt measure and had lesser correlation with other measures in our analyses. Thus, we only used the willingness to adopt this measure.

#### Perceived Value

How would you rate the importance or value of the technology that was just presented to you (1 being not at all important, 5 being somewhat important, and 9 being extremely important)?

#### Perceived Effort

How much effort do you think you would have to put into learning and mastering the technology that was just presented to you (1 being none, 5 being some, and 9 being a lot)?

#### Confidence in Using the Technology

How confident are you that you have the ability to learn and master the technology that was just presented to you (1 being not at all confident, 5 being somewhat confident, and 9 being extremely confident)?

#### Help With Technology

How much help would you need from family and friends to learn and master the technology that was just presented to you (1 being none, 5 being some, and 9 being a lot)?

#### Quality of Life

Think about the things that are most important to you that contribute to your quality of life. How much can the technology just presented to you help improve your quality of life (1 being not at all, 5 being some, and 9 being a lot)?

#### Concern With Privacy

How worried or concerned are you about privacy and trust issues associated with the technology just presented to you (1 being not at all worried, 5 being somewhat worried, and 9 being extremely worried)?

#### Willingness to Pay

Select how much you would be willing to pay to own the technology just presented to you. When making your selection, do not include any ongoing or recurring costs for services or subscriptions associated with the technology. (Participants could choose from nothing to more than US $100, with 10 categories in between these extremes).

### Discounting the Investment of Time to Learn the Technologies

The experimenter, following a script, guided the administration of 2 complementary instruments designed for assessing discounting behavior that were presented to the individual participants on the laptops provided to them. First, the Time Allocation to Attain Skill instrument was used for participants to indicate the amount of time (in hours and minutes) that they would be willing to spend to achieve a certain level of skill on the specified technology. Five levels of skill—basic, moderate, intermediate, advanced, and mastery—were defined, and training was given to clarify differences among these skill levels. Participants responded either yes or no regarding their desire to attain the next level of skill in the technology. If their choice was “yes,” they were also asked to indicate the additional amount of time they would be willing to invest to achieve that skill level; however, they could choose to stop at any level if they did not desire to acquire any further skill in that technology.

After completing this instrument for each of the 5 technologies, the participants completed the Assigning Importance to a Skill Level instrument. Using their laptops, they were instructed to rate the importance of attaining the desired skill levels that they had previously indicated for each of the 5 technologies on a scale that ranged from 1 to 10 (1 indicated no importance, 5 indicated average importance, and 10 indicated extremely important). Participants were cued (by the computer interface) to assign importance values only for those skill levels for which they indicated that they were willing to invest time to attain.

These 2 instruments enabled the collection of data for determining the degree to which participants discounted the time they were willing to invest to acquire skills for each of the 5 technologies [27]. In addition, the level of skill desired, defined as the highest level of skill participants wished to attain for each technology (ranging from 1 for basic skills to 5 for a skill level of mastery) was also used as a measure as desire to acquire greater skills was believed to be indicative of willingness to adopt the technology.

### Additional Instruments

#### Cognitive Test Battery

Participants were administered the Trail Making Tests A and B [33], which measure overall cognitive functioning; Digit Span, forward and backward [34], which measures working memory; the Shipley Vocabulary test [35], which measures crystallized and fluid cognitive ability; and the Multidimensional Aptitude Battery [36] test, which measures life knowledge.

#### Self-assessment of Abilities

Participants completed an 8-item rating scale, adapted from Ackerman and Wolman [37], which was used to assess their self-appraisal of the following abilities on a 9-point scale (1=very low ability; 9=very high ability): vocabulary, comprehension, numeric ability, memory, learning ability, problem-solving and reasoning, detection, and grasping and manipulative skill.

#### Openness to New Experiences

Participants answered 2 questions from the Ten-Item Personality Inventory [38] related to traits associated with being open to new experiences.

#### Perceptions of Aging

Participants answered the 10-item Attitudes Toward Age-Related Change [39], which is divided into two 5-item sections measuring the perceptions of positive and negative aspects of aging. Each item ranges from 1 to 5 or not at all to very much.

#### Technology Readiness

Participants completed the Technology Readiness Index, a 16-item questionnaire that uses a 5-point Likert scale designed to determine an individual’s predisposition to adopting new technologies [40] and thus capture potentially important attitudes related to technology acceptance. It comprises 2 positive dimensions, optimism (belief that technology increases control, flexibility, and efficiency) and innovativeness (one’s view of being a “technology pioneer”), and 2 negative dimensions, discomfort (a tendency to being uncomfortable with or overwhelmed by technology) and insecurity (a general feeling of skepticism or fear toward technology). Participants were asked to what extent they agree or disagree with 16 statements across the 4 dimensions.

#### General Technology Use Survey

This instrument was divided into three sections: (1) a section which asked participants about their access to and use of computer technologies, such as desktops, laptops, and tablets, smartphones, internet, and email; (2) a section comprised of 4 questions to which participants indicated, on a 9-point scale (1=very low; 9=very high), their degree of basic computer technology skill (eg, the ability to easily use the equipment associated with basic computer technologies such as a keyboard or a mouse); internet and email skill or knowledge; computer programs knowledge; and computer applications knowledge (eg, about different applications or “apps” on a computer or smartphone and how to use them); and (3) a section related to participants’ needs for assistance and support in use of technology. This last section consisted of 2 questions to which participants responded, using a 9-point scale (1=none of the time, 5=some of the time, and 9=all the time), how often they needed assistance to help them learn and master a new technology and how often someone was available to them to learn and master a new technology. In addition, participants were asked to check off items indicating who they relied on for help learning to use a new technology and who they listen to for advice and recommendations when considering whether to use a new technology.

Table 2 summarizes the measures used in this study as well as the instruments from which they were obtained.

**Table 2:** Variables collected in the study.

Measure	Type of variable^a^	Instrument
Age	Individual (continuous)	Demographics questionnaire
Perceived overall health	Individual (scale value: 1-5)	Demographics questionnaire
Willingness to adopt the technology	Technology (scale value: 1-9)	Technology Rating Questionnaire
Perceived value of the technology	Technology (scale value: 1-9)	Technology Rating Questionnaire
Perceived effort to learn the technology	Technology (scale value: 1-9)	Technology Rating Questionnaire
Self-confidence in ability to learn and use the technology	Technology (scale value: 1-9)	Technology Rating Questionnaire
Perceived help needed to learn the technology	Technology (scale value: 1-9)	Technology Rating Questionnaire
Quality of life improvement from the technology	Technology (scale value: 1-9)	Technology Rating Questionnaire
Privacy or trust issues with the technology	Technology (scale value: 1-9)	Technology Rating Questionnaire
Willingness to pay to own the technology	Technology (categorical)	Technology Rating Questionnaire
Relative comparisons in adopting the technologies	Technology (a set of relative weights of each technology)	Paired Comparison Ratings Instrument
Discounting rate	Technology (continuous)	Time Allocation to Attain Skill Instrument
Level of skill desired	Technology (scale value: 1-5)	Time Allocation to Attain Skill Instrument
Overall cognitive functioning	Individual (test score)	Cognitive Test Battery (Trail Making Tests A and B)
Working memory	Individual (test score)	Cognitive Test Battery (Digit Span)
Crystallized and fluid cognitive ability	Individual (test score)	Cognitive Test Battery (Shipley Vocabulary)
Life knowledge	Individual (test score)	Cognitive Test Battery (Multidimensional Aptitude Battery)
Self-assessment of cognitive abilities	Individual (average score; item scale value: 1-9)	Self-Assessment of Abilities Questionnaire (6 of 8 items)
Openness to new experiences	Individual (average score; item scale value: 1-7)	Ten-Item Personality Inventory (2 of the items)
Perceptions of aging: gain	Individual (sum score; item scale value: 1-5)	Perceptions of Aging (5 of 10 items)
Perceptions of aging: loss	Individual (sum score; item scale value: 1-5)	Perceptions of Aging (5 of 10 items)
Technology readiness: optimism, innovativeness, discomfort, and insecurity	Individual (average total and subscale scores; item scale value: 1-5)	Technology Readiness Index Questionnaire
Self-assessment of technology skill	Individual (average of 5 items; item scale value: 1-9)	General Technology Use Survey
Needs or availability for technology assistance support	Individual (average of items; item scale value: 1-9)	General Technology Use Survey
General tech experience	Individual (sum score of yes or no to having used 5 technologies)	General Technology Use Survey

^a^Technology variables were collected for each of the 5 technologies; individual variables were collected once.

### Analytic Approach

As noted earlier, because of the large number of variables and the difficulty of specifying a predictive model a priori with such a large number of parameters and potential collinearity, we adopted a systemic exploration analytic method based on machine learning techniques, the k-fold cross-validation regression technique, to derive our model of willingness to adopt the technologies. We chose this technique as it helps reduce model overfitting and provides a better estimate of how our derived model would perform in general, beyond the data generated by our sample [41].

Initially, because of missing data (<1% of total observations), for some of the variables, we used multiple imputation to create 20 different complete data records for each of the 187 participants. For each participant, each of these data records contained complete data for all variables, with the prior missing data replaced by imputed values. Thus, we generated 20 data records for each participant, each with 20 different imputed values for each variable with missing data. Although typically 5 imputations for missing data are considered sufficient, we opted to be conservative and instead created 20 different data records for each participant for reasons involving our variable selection method explained in the following paragraphs.

Following imputation and the generation of 20 data records, the next step was to identify the set of variables that would best predict our main dependent variable, willingness to adopt the technologies. We tested each data record with a k-fold cross-validation regression program as implemented in *glmnet* for the R statistical environment [42]. This program uses a penalized regression technique to handle collinearity and is consistent with the techniques of Ridge regression and Lasso regression. The k-fold cross-validation estimates model parameters on part of the data and then validates those parameters on a separate subsample not used to estimate the parameters. The program attempts to find the set of parameters that best fits the separate subsample while varying the lambda penalty (a value that shrinks regression parameters toward 0) from 0 (equivalent to a Ridge regression) to 1 (equivalent to a Lasso regression).

Once the 20 k-fold cross-validated regressions were computed, we recorded the number of times across the 20 data records each variable was predictive and the average parameter value each time it was predictive. The criterion we adopted was that variables would be selected for further exploration if they were found to be significant in at least half (10/20, 50%) of the regression models, as this would result in a model that is more generalizable and less biased. We viewed variables that were predictive in all 20 data records as more likely to be producing a replicable effect then those that were not predictive in all 20 data records. We also viewed those variables that were predictive more often than not as more likely to replicate in future research than those that were not predictive more often than not, and we encourage the reader to use the same heuristic. Although we report model data for those variables that were selected by the model in <10 of the data records based on the previous reasoning, we do not provide an interpretation of the parameters for these variables.

We then conducted a series of regression analyses for the purpose of enhancing our understanding of the variables predicting willingness to adopt (eg, understand the effect sizes associated with the variables), using the set of variables selected by the k-fold cross-validated regressions.

However, consistent with our study objectives in understanding the facilitating and inhibitory roles of various variables in influencing technology adoption willingness, we were also interested in understanding if the strongest predictors of willingness to adopt technologies were potentially mediating the relationships of other variables (eg, crystallized intelligence and technology readiness) related with willingness to adopt technologies. For example, the findings from our analysis of willingness to adopt indicated that it was most strongly predicted by the participants’ ratings of improvement in quality of life from the technology, perceived value of the technology, and confidence in using the technology. To examine the potential mediating role of these 3 variables, we again performed k-fold cross-validated regressions in *glmnet*, this time with the ratings of quality of life, perceived value, and confidence serving as the dependent variables.

Having identified a set of predictors for each of the 3 variables using cross-validation, we again conducted separate regression analyses on each of the 3 primary predictors of willingness to adopt technologies, once again with the goal of getting the more intuitive output with effect sizes and statistical significance. For example, as an illustration of this analytic process, in the k-fold regressions, crystallized ability had no direct relationship with willingness to adopt. However, it was negatively related to quality of life and positively related to confidence using the technology in the k-fold regression of those 2 variables.

## Results

### Overview

The study sample included 187 adults aged 65 to 92 (mean 74.1, SD 6.3) years, who were primarily women (145/187, 77.5%); diverse in age, with 41.1% (77/187) of the participants aged ≥75 years; and diverse in ethnicity and race—21.3% (40/187) of the participants identified as Hispanic and 35.8% (67/187) identified as Black or African American. Most participants (157/187, 83.9%) reported having at least some college education, 82.9% (155/187) reported being retired, and 89.8% (168/187) self-reported their health as at least good. Table 3 includes descriptive statistics for the sample demographics. Table 4 shows the results of the k-fold cross-validated regressions with the parameter estimate and the number of multiply imputed data sets for the parameter that was selected as predictive; again, we urge caution in interpreting variables that were not selected in most of the models. For each of the 20 models, the following 4 variables were predictive of higher ratings of willingness to adopt the technologies: higher ratings of perceived value of the technologies, higher ratings of perceived improvement in quality of life by the technologies, higher rating of confidence using the technologies, and greater technology experience. In addition, across all 20 models, higher ratings of perceived help needed to learn the technologies were predictive of lower ratings of willingness to adopt the technologies.

**Table 3:** Demographics of the sample (n=187).

Variable			Values
Tech experience, mean (SD)			3.87 (1.50)
Age (years), mean (SD)			74.11 (6.33)
General health, mean (SD)			3.43 (0.85)
Gender (women), n (%)			145 (77.5)
**Education, n (%)**			
	High school or less	22 (11.8)	
	Some college or associates	51 (27.4)	
	Bachelor’s degree	48 (25.8)	
	Postgraduate	58 (31.2)	
	Vocational	7 (3.8)	

**Table 4:** Results of 20 multiple imputed cross-validated regression.

	Willingness to adopt technology			Quality of life from technology			Perceived value of technology			Confidence using technology	
	M^a^	β	M		β	M		β	M		β
Intercept	20	−.11	20		3.19	20		4.55	20		6.34
Tech readiness optimism	—^b^	—	20		.11	20		.11	20		.02
Tech readiness innovativeness	15	.01	—		—	—		—	—		—
Tech readiness insecurity	14	.00	2		.00	—		—	—		—
Positive tech readiness	18	.01	19		.01	—		—	7		.00
General tech experience	20	.06	—		—	—		—	20		.06
Gender (women)	8	−.06	—		—	—		—	0		—
Education	—	—	—		—	—		—	20		.03
General health	—	—	11		−.04	—		—	—		—
Self-assessed comprehension	—	—	—		—	—		—	19		.01
Self-assessed learning ability	—	—	—		—	—		—	11		.00
Self-assessed applying new knowledge	—	—	—		—	—		—	20		.03
Self-assessed problem-solving or reasoning	—	—	—		—	—		—	20		.02
Self-assessed detection	—	—	—		—	—		—	19		.02
Cognitive abilities: fluid	—	—	1		−.02	—		—	—		—
Cognitive abilities: crystalized	—	—	18		−.08	7		−.02	20		.14
Perceptions of aging: gains	—	—	12		.01	1		.00	—		—
Openness to experience	—	—	—		—	—		—	13		.01
Help with technology	20	−.03	—		—	—		—	20		−.20
Confidence using technology	20	.15	N/A^c^		N/A	N/A		N/A	N/A		N/A
Concern with privacy of technology	—	—	1		.00	—		—	—		—
Perceived effort of technology	—	—	20		.11	20		.04	—		—
Perceived value of technology	20	.54	N/A		N/A	N/A		N/A	N/A		N/A
Quality of life from technology	20	.24	N/A		N/A	N/A		N/A	N/A		N/A
Discounting parameter^d^	—	—	15		−.27	—		—	—		—

^a^M: number of models in which the parameter was included.

^b^—: variable was not selected as predictive in any data record for this dependent variable.

^c^N/A: not applicable.

^d^Tech readiness discomfort, negative tech readiness, age, self-assessed vocabulary, self-assessed numeric, self-assessed, memory, self-assessed grasping, and perceptions of aging losses were included in the model; however, they are not presented because they were not predictive in any model computed.

### Factors Effecting Willingness to Adopt Technology

The k-fold analyses on willingness to adopt technologies found that the following variables were predictive in ≥10 of the 20 imputed data sets: perceived value of the technologies, perceived improvement of quality of life by the technologies, confidence using the technologies, perceived help needed with the technologies, the innovativeness component of technology readiness, the insecurity component of technology readiness, positive technology readiness, and technology experience.

In the multiple regression analysis, these 8 variables selected by cross-validation explained a large amount of variance (*F*_8,178_=59.7, *P*<.001, *R*^2^=0.73). Five of these variables were significantly predictive in the multiple regression. For each statistically significant predictor, we report first the regression parameter and then the zero-order correlation. The zero-order correlation is reported to allow the reader to see how the predictor relates to the target variable, in this case willingness to adopt technology, without the rest of the variables and thus how the other variables included in the multiple regression are altering the effect size. Of these, the perceived value of the technologies was the strongest predictor (β=.60, *P*<.001; *r*=0.77, *P*<.001), followed by perceived improvement in quality of life by the technologies (β=.32, *P*<.001; *r*=0.71, *P*<.001). Confidence using the technologies was the next strongest predictor (β=.15, *P*=.009; *r*=0.45, *P*<.001), followed by perceived help needed with the technologies (β=−.10, *P*=.02; *r*=0.17, *P*<.001) and then technology experience (β=.12, *P*=.03; *r*=0.30, *P*<.001). Therefore, individually, perceived value and improvement in quality of life accounted for >50% of the variance in willingness to adopt the technologies, confidence accounted for 20%, and technology experience accounted for 9%. It is also notable that in this model of only 8 predictor variables, the technology readiness variables were not significant, although they were selected in over half of the models as improving the models’ predictive accuracy.

### Factors Effecting Perceived Value of Technologies

As noted, given the strong relationship of willingness to adopt technologies with perceived value, improvement in quality of life, and confidence in using the technologies, we hypothesized that these variables were potentially mediating the relationships of other important variables. The findings from the k-fold cross-validation analyses indicated that across all 20 imputed data records, higher scores in the optimism component of the technology readiness scale and higher scores in perceived effort needed to learn the technologies were positively related to perceptions regarding the impact of the technologies on perceived value.

The results of the multiple regression analyses indicated that these 2 variables together strongly positively predicted perceived value (*F*_2,184_=29.88, *P*<.001, *R*^2^=0.25). The individual parameters were β=.19 (*P*<.001) and *r*=0.43 (*P*<.001) for the optimism component of technology readiness and β=.21 (*P*<.001) and *r*=0.25 (*P*=.001) for perceived effort required by the technologies.

### Factors Effecting Quality of Life From Technologies

With respect to perceptions of improvements in quality of life from the technologies, the models across all 20 data records selected the optimism component of technology readiness and perceived effort required by the technology, with higher values in both being associated with higher quality of life from adopting the technologies. Selected by most models but not all models as predicting higher levels of quality of life from the technology were higher levels of positive technology readiness, lower crystallized intelligence, lower values in the discounting of the technologies, higher perception of gains with aging, and lower health. The follow-up multiple regression analysis on this set of 7 variables indicated that this set of variables explained a large amount of variance in perceived improvement in quality of life (*F*_7,179_=13.08, *P*<.001, *R*^2^=0.33). Of these variables, 3 were significantly predictive of perceived improvement in quality of life in the multiple regression. Of these, the 2 strongest relationships were higher perceived effort required by the technology (β=.18, *P*=.001; *r*=0.30, *P*<.001) and the positive aspect of technology readiness (β=.14, *P*<.001; *r*=0.41, *P*<.001). Subsequently, lower discounting predicted higher perceived improvement in quality of life (β=−.86, *P*=.03; *r*=0.24, *P*=.001).

### Factors Effecting Confidence in Using Technologies

With respect to confidence in using the technologies, the variables predicting higher confidence using the technology found by a k-fold cross-validation regression were higher crystallized intelligence, technology experience, self-assessed applying of new knowledge, self-assessed problem-solving or reasoning, general education, scores on the optimism component of technology, and needing less help with technologies. Selected by most models but not all models as predicting higher confidence using the technologies were the following variables: higher self-assessed comprehension, higher self-assessed detection, higher openness to experience, and higher self-assessed learning ability. Interestingly, however, although the multiple regression on this set of variables predicting higher confidence explained a large amount of variance (*F*_11,175_=13.14, *P*<.001, *R*^2^=0.45), likely due to collinearity, only 2 variables were predictive in the multiple regression. Lower scores in perceived help needed with the technologies (β=−.25, *P*<.001; *r*=0.31, *P*<.001) and higher scores in the optimism component of technology readiness (β=.07, *P*=.02; *r*=−0.54, *P*<.001) were predictive of higher confidence in using the technologies.

## Discussion

### Summary

The importance of identifying and understanding the factors that contribute to people adopting technology stems from the considerable benefits that numerous powerful and easily accessible technologies can potentially provide. For many older adults, these benefits may be especially beneficial, as these individuals may be experiencing cognitive and physical limitations or declines in health states related to social isolation or declines in functional abilities, which could possibly be circumvented or even overcome with the aid of technologies. However, research related to technology adoption for this population of adults, not unlike prior studies on technology adoption for younger populations, has been limited to identifying factors based on adults’ responses to questionnaire items directed at general technology adoption. In this study, we implemented an innovative approach referred to as the TAP, which was intended to capture study participants’ willingness to adopt each of the 5 technologies included in this study. This methodology enabled participants to assess their perceptions related to specific technologies by providing them, through a series of formal presentations, basic information associated with a set of technologies that were believed to provide unique benefits to the health and well-being of older adults [1]. In addition to being able to assess the willingness to adopt technology within the context of concrete technologies, we incorporated an empirical experimental perspective that included the administration of various tools intended for assessing other variables that were hypothesized to either facilitate or inhibit the behavioral intentions underlying the primary dependent variable, the willingness to adopt technology, and a machine learning approach in the selection of predictor variables coupled with regression analysis for assessing the possible mediating roles of those predictors. Furthermore, our sample included a diverse sample of 187 older adults that included those in the older age cohort.

### Implications for Models of Technology Adoption

A robust study result was the finding that higher ratings on the following 4 variables predicted higher ratings of willingness to adopt the technologies, and higher ratings on a fifth variable predicted lower ratings of willingness to adopt the technologies. These variables consisted of perceived value of the technologies, perceived improvement of quality of life by the technologies, perceived confidence in using the technologies, higher scores on technology experience, and higher ratings of perceived help needed with the technologies (which was predictive of lower ratings of willingness to adopt the technologies). The results for perceived value, improvement in quality of life, and confidence are consistent with past studies of the TAM [10,11] and STAM [16,17], as well as with a study that implemented the TAP method [1]. Davis argued that intention to use technology was determined by perceived ease of use and perceived usefulness. The largest predictors that we found, perceived value and perceived improvements in quality of life, could be interpreted as corresponding to the construct of perceived usefulness, and our next strongest predictors, confidence in using the technologies and perceived help needed with the technologies, could be construed as relating to perceived ease of use. In the STAM [16,17], which focused on older adults (aged >55 years), the factors found to predict general technology acceptance were relatively broad, such as self-efficacy, gerontechnology anxiety, and health and ability characteristics, and could be viewed as related to the predictor of confidence in using the technologies.

Unlike the STAM and TAM, this study was based on a concurrent perspective which used presentations of concrete contexts of specific technologies as a basis for providing appraisals related to willingness to adopt technology. Therefore, we suggest that the variables perceived value and perceived improvements in quality of life, although clearly related conceptually, are distinctive predictors. Because participants were given the opportunity to consider the present adoption of the technology, they may have been able to dissociate general usefulness or value from more specific ways in which the quality and independence of their lives could be influenced by the technologies. Thus, we also suggest that the perceived value associated with a technology captures an appraisal more closely linked to the general appraisal of a technology as useful, whereas perceived improvements in quality of life represent appraisals that enable technologies to be more differentiable based on the extent to which they might positively modify one’s life. Similarly, although self-efficacy resembles the degree of confidence in one’s ability to learn and master the technology (the variable used in our study), the self-appraisals of confidence collected following presentations on each technology may be more dependent on the nature of the specific technology than on a more general self-assessed state of self-efficacy. Greater emphasis on current confidence in using a specific technology, as opposed to the more general retrospective assessment of one’s self-efficacy, is more consistent with the perspective of Lee and Coughlin [43] on the importance of confidence in older adults’ adoption of technology and, as will be discussed later, provides a more direct bridge to strategies intended to market technologies for older users.

The use of concrete contexts as a basis for assessing technology adoption also likely influenced the finding that a greater perceived need for help was found to reduce willingness to adopt the technology, as this information would provide the participant with a greater understanding of the predicaments in which they might find themselves when attempting to adopt or use a specific technology without available support. Finally, the positive relationship between general technology experience and willingness to adopt the technologies, although not related to a self-appraisal linked to the presentation of a specific technology, is also informative, as it provides for an assessment of self-efficacy as it pertains to technology use.

However, to establish both a more comprehensive understanding of factors predicting technology adoption for older adults and to develop strategies for marketing technologies to increase the likelihood of their adoption by these users, other variables need to be considered that could have facilitated or inhibited the possible mediating roles the primary predictors discussed above had on willingness to adopt the technologies. The most robust finding (ie, across all 20 imputed data records) was that for both perceived value and improvements in quality of life, the optimism component of the technology readiness scale and perceived effort needed to learn the technologies each had a positive influence on perceived value and improvements to quality of life from the technology. Optimism in technology readiness (the belief that technology increases control, flexibility, and efficiency) appears to be the more critical of the 2 positive dimensions on this scale, and within the context of considering adopting specific technologies (such as those considered in this study), likely to represent a powerful attitudinal perspective to the behavioral intention to adopt a technology and thus underly ratings of perceived value and improvements to quality of life.

Perhaps less intuitive to the possible mediating roles of these 2 predictors of willingness to adopt technologies is the positive relationship the perceived effort needed to learn the technologies had with them. Earlier, we had hypothesized that increased perceived cognitive effort needed to learn technologies would negatively impact willingness to adopt, as people tend to minimize cognitive effort. However, the overall findings may suggest that if older users demonstrate optimism in their technology readiness attitudes, this may override the tendency to avoid investing effort in learning, especially if the technologies are perceived as capable of providing improvements to quality of life.

Other variables found in most (but not all) of the 20 imputed data sets that significantly influenced improvements in quality of life included crystallized intelligence, discounting of the technologies, and perceived health, with lower values for each of these variables positively associated with improvements in quality of life and willingness to adopt the technologies. For this study sample, increased age was found to be associated with decreased discounting [27], suggesting that the greater time older adults were willing to invest to achieve higher skill levels in these technologies may be linked to their perceptions that attaining these higher-level skills could translate to improvements in the quality of their lives. The weaker relationships that were found between lower levels of crystallized intelligence and lower perceived health on perceptions of increased improvements to quality of life and willingness to adopt the technologies are less understood; they may suggest self-awareness by these participants of the need to compensate for these lower cognitive and health levels through technologies that could potentially benefit their health and well-being.

Expectedly, confidence in having the ability to learn the technologies as a positive predictor of willingness to adopt them was predicted by higher self-assessments of abilities such as comprehension and learning abilities, higher scores in the optimism component of the technology readiness scale, and lower ratings of perceived need for help to learn to use the technologies. Taken together with the findings for the other 2 main predictors of willingness to adopt the technologies, perceived value, and improvements in quality of life, some strategies for inducing adoption of technologies by older adults are suggested. For example, in marketing these technologies and developing methods for instruction on their use, emphasis should be given to very specific ways the technology can benefit independence and quality of life and how efficient these technologies can be in meeting these goals. Although designing technological products that are easy for older adults to use is critical [44], if these designs are usable, older adults are likely to not be deterred if cognitive effort, within reason, is needed to learn to use the technologies. In addition, they may be willing to invest additional time to attain higher levels of mastery, provided the benefits of the technology are evident. Messages that promote optimism in technology are also recommended as they provide the basis for positive underlying attitudes.

### Limitations

The main limitation of this study is that it is a concurrent, cross-sectional study, and participants did not have the opportunity to actually engage with the technologies. Thus, although a great deal of attention was paid to familiarize the participants with each technology so that participants could provide responses that were as accurate as possible about their willingness to adopt each of the technologies that were presented, collecting prospective real-world data on actual use patterns would be preferred, but this was beyond the scope of this study.

However, this study overcame many limitations associated with retrospective, questionnaire-based data by providing more realistic contexts for assessing technology adoption and a larger array of variables informed by an understanding of the cognitive capabilities and limitations of older adults. In addition, 2 major strengths of the study are the comprehensive explanation and walkthrough of the target technology, which we feel is necessary for participants to have an accurate understanding of the technologies, and our rigorous measurement of their perceptions and many related constructs. However, these comprehensive explanations and measurements limited us to only the 5 technologies selected. Although we feel the findings of our study likely generalize beyond these specific technologies, that must be confirmed by future research.

In addition, because the study used only 5 technologies, we were not able to study the differences in technology adoption between technologies within the scope of this project. Future research should expand upon the technologies used here and potentially look at heterogeneity in what predicts technology adoption between different types of technologies. In addition, the participants self-selected into the study, and the study advertisement stated that the study was about technology and might require participants to travel to the University of Miami-Miller School of Medicine or Weill Cornell Medicine. This may have impacted the sample recruitment. In this regard, the sample was likely healthier, more interested in technology, and more educated and thus not representative of the diverse population of older adults living independently. In addition, we could not include people with cognitive deficits because of the nature of the study requirements.

### Conclusions

This study provided a conceptual basis for identifying variables that could influence older adults’ willingness to adopt technology and used a concurrent framework whereby participants’ appraisals regarding their willingness to adopt technology were made within the context of exposure to 5 exemplar technologies with potential benefits to older populations. The analytic approach taken enabled direct predictors of willingness to adopt technology, as well as variables that had inhibitory and facilitating influences on these predictors to be determined. Future research examining the issues of technology adoption could benefit from the methods used in this study and examining the complex patterns of relationships found. On the basis of the variables identified as important in this study, in future studies, it should be easier to select the number of variables for investigation and further expand our causal model. However, the ultimate criterion for a model of technology acceptance among older adults is the longitudinal measurement of the use of technology in the naturalistic environment, which for many reasons remains a challenging problem.

## Abbreviations

STAM: Senior Technology Acceptance Model
TAM: Technology Acceptance Model
TAP: Technology Assessment Procedure
UTAUT: Unified Theory of Acceptance and Use of Technology

## Data Availability

Data are available upon request. The authors are currently merging data sets on technology adoption across the Center on Aging at the University of Miami and the Center for Aging and Behavioral Research at Weill Cornell Medicine.
